# Pathological study of sternal osteomyelitis after median thoracotomy—a prospective cohort study

**DOI:** 10.1007/s00423-023-02926-0

**Published:** 2023-05-11

**Authors:** Olimpiu Bota, Jessica Pablik, Feras Taqatqeh, Maxime Mülhausen, Klaus Matschke, Adrian Dragu, Stefan Rasche, Kevin Bienger

**Affiliations:** 1 University Center for Orthopedics, Trauma and Plastic Surgery, Faculty of Medicine Carl Gustav Carus, TU Dresden, Fetscherstraße 74, 01307 Dresden, Germany; 2 Institute for Pathology, Faculty of Medicine Carl Gustav Carus, TU Dresden, Dresden, Germany; 3 Department of Cardiac Surgery, University Heart Center, TU Dresden, Dresden, Germany; 4 Surgical Intensive Care Unit, Faculty of Medicine Carl Gustav Carus, TU Dresden, Dresden, Germany

**Keywords:** Deep sternal wound infection, Sternal osteomyelitis, Cardiac surgery, Thoracic surgery

## Abstract

**Purpose:**

Osteomyelitis of the sternum may arise either as a primary condition or secondary to median thoracotomy after cardiac surgery, with the latter being decidedly more frequent. Deep sternal wound infections appear as a complication of median thoracotomy in 0.2 to 4.4% of cases and may encompass the infection of the sternal bone. To date, there are no exhaustive histopathological studies of the sternal osteomyelitis.

**Methods:**

Our work group developed a surgical technique to remove the complete infected sternal bone in deep sternal wound infections. We therefore prospectively examined the en bloc resected sternal specimens. Seven standard histological sections were made from the two hemisternums.

**Results:**

Forty-seven sternums could be investigated. The median age of the patients in the cohort was 66 (45–81) years and there were 10 females and 37 males. Two methods were developed to examine the histological findings, with one model dividing the results in inflammatory and non-inflammatory, while the second method using a score from 0 to 5 to describe more precisely the intensity of the bone inflammation. The results showed the presence of inflammation in 76.6 to 93.6% of the specimens, depending on the section. The left manubrial sections were more prone to inflammation, especially when the left mammary artery was harvested. No further risk factors proved to have a statistical significance.

**Conclusion:**

Our study proved that the deep sternal wound infection may cause a ubiquitous inflammation of the sternal bone. The harvest of the left mammary artery may worsen the extent and intensity of infection.

## Background

**Fig. 1:**
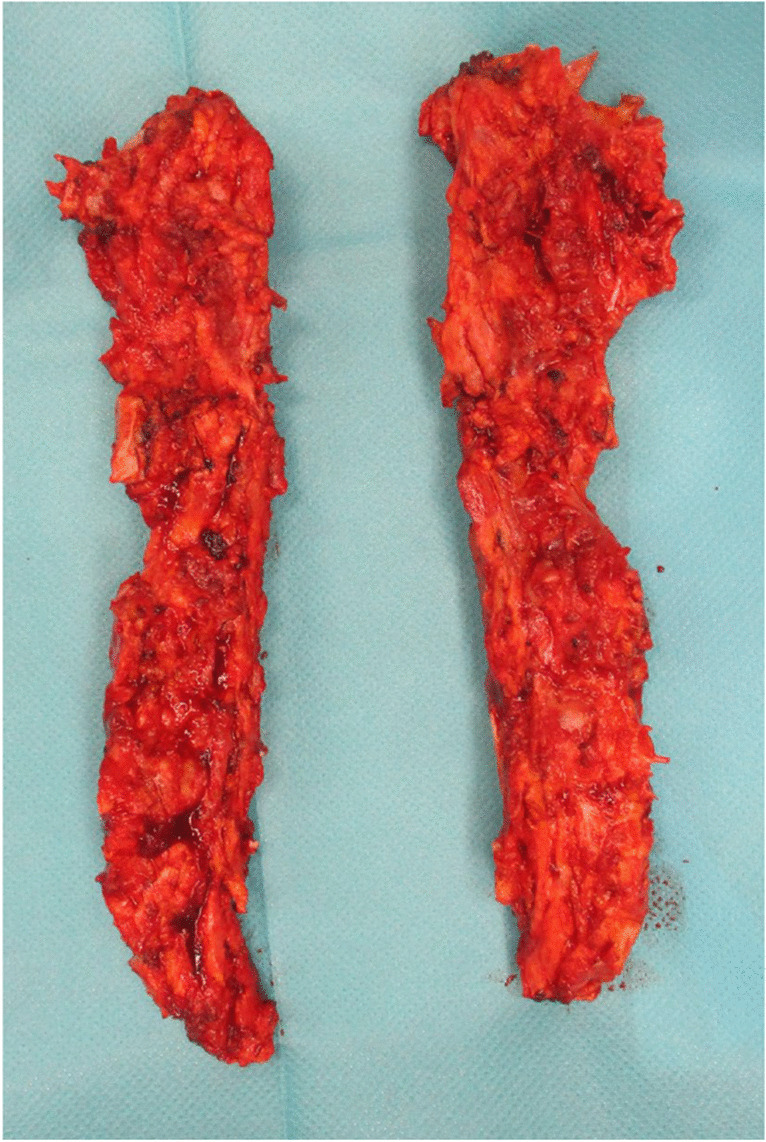
Hemisternums after en bloc resection

Osteomyelitis of the sternum may arise either as a primary condition or secondary to median thoracotomy after cardiac surgery. The primary sternal osteomyelitis (SO) is rare and usually develops due to hematogenic dissemination. Different types of microorganisms have been found to cause this disease, from the common *Staphylococcus aureus* to typical and atypical Mycobacteria [1, 2]. The secondary SO is decidedly more frequent. This condition complicates the median thoracotomy interventions for cardiac surgery in 0.2–4.4% of the cases, being part of the larger notion of deep sternal wound infections (DSWIs), where next to the bone, the mediastinal soft tissues are infected [3, 4]. When a SO has been diagnosed through microbiological and histopathological probes, the treatment usually comprises the surgical excision of the infected, necrotic bone (sequestrum) and the flap coverage after infection treatment using negative pressure wound therapy [5]. To eradicate the infection completely and safely, instead of a piecemeal sternal resection, a complete sternectomy can be performed. Our study group has established an operative technique, in which the whole sternum is being resected en bloc (Fig. 1). The resected parts, either in one piece if the two sides are united by scar, or in two pieces if the sternum is still separated after the thoracotomy, are sent to the histopathological examination.

The goal of our study was to present the pathological changes in SO after median thoracotomy and identify the risk factors which contribute to the downfall of the sternal bone. Our working hypothesis was that there are different levels of infection in the different histological sections of the sternal bone depending on comorbidities and surgical technique.

## Material and methods

We prospectively examined the en bloc resected sternums in 47 patients operated at our institution for deep sternal wound infection between December 2018 and September 2021. The indication for a radical sternectomy was established interdisciplinarily between the cardiac and the plastic surgeon, when a secondary closure of the thoracotomy wound was not feasible, due to relapsing infections or to sternal bone destruction. After debridement, the patients received negative pressure wound therapy and the wounds were secondarily closed with pedicled latissimus dorsi musculocutaneous flaps or pectoralis major muscle flaps.

**Fig. 2:**
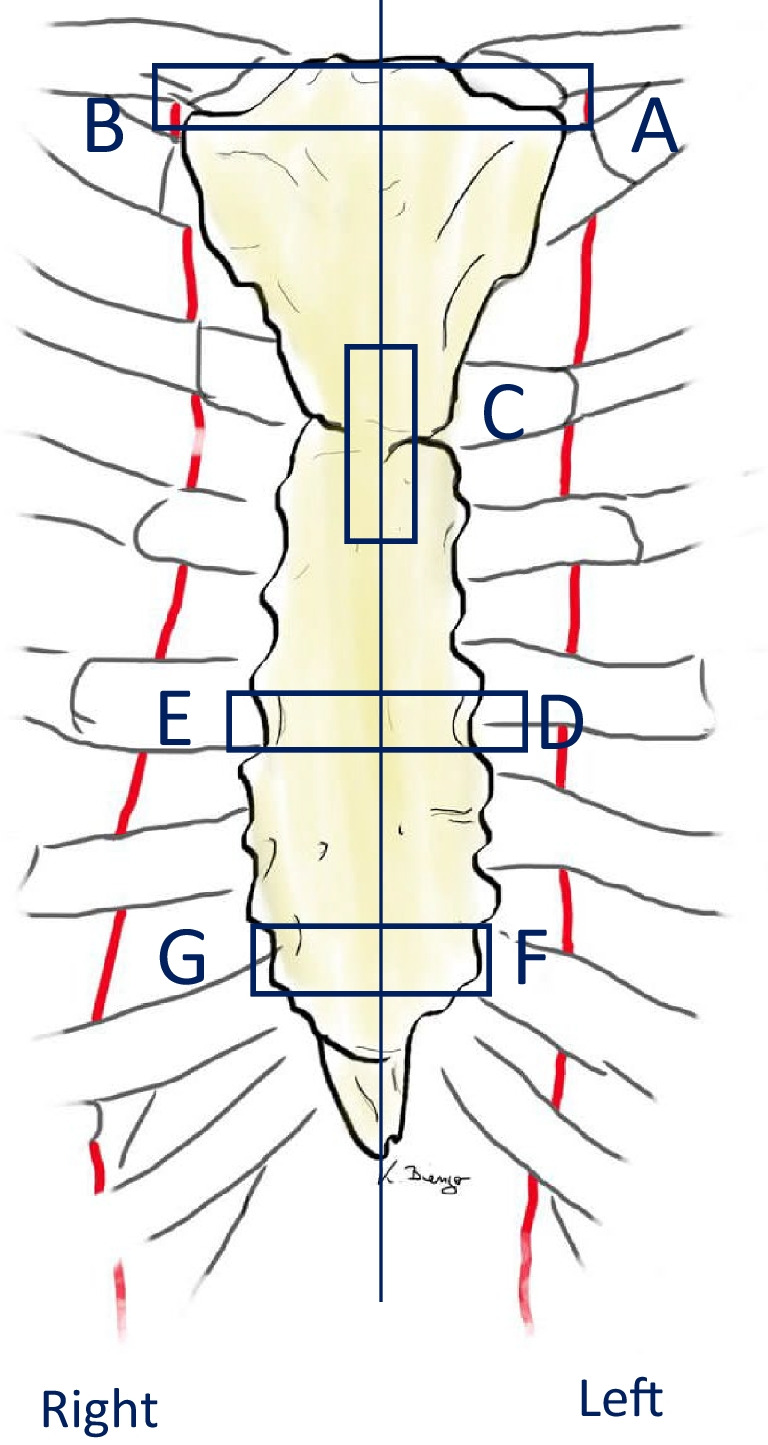
Histological sections

Seven histopathological sections of the bone were performed according to Fig. 2. The sections were then examined by a board-certified pathologist.

The microscopical examination showed different degrees of inflammation within the sternal bone. For the statistical analysis, two methods were applied. The first model divided the results in inflammatory and non-inflammatory, while the second model used a score to describe more precisely the intensity of the bone inflammation (Table 1). The statistical analysis was performed using IBM SPSS Statistics for Windows (IBM Corp. Released 2021, Version 28.0. Armonk, NY: IBM Corp). For the nominal variables from the first statistical model, contingency tables were used and the Fisher’s exact test was performed. The scores in the second statistical model were analyzed usings Cohen’s Kappa coefficient. The nominal values in this second model were analyzed using contingency tables and the chi square test (*χ*^2^) A statistical significance was considered when *p*<0.05.

**Table 1:** Scoring system to evaluate the degree of bone inflammation

Inflammation type	Model 1	Model 2—scoring
Purulent-sequestering	Inflammation	5 points
Purulent-sequestering/chronic-granulating (Fig. 5)	Inflammation	4 points
Chronic-granulating	Inflammation	3 points
Chronic-granulating/fibrosing	Inflammation	2 points
Fibrosing (Fig. 6)	Inflammation	1 point
No Inflammation	No inflammation	0 points

The study was approved by the ethics committee of our institution (*EK 387082020*).

## Results

**Fig. 3:**
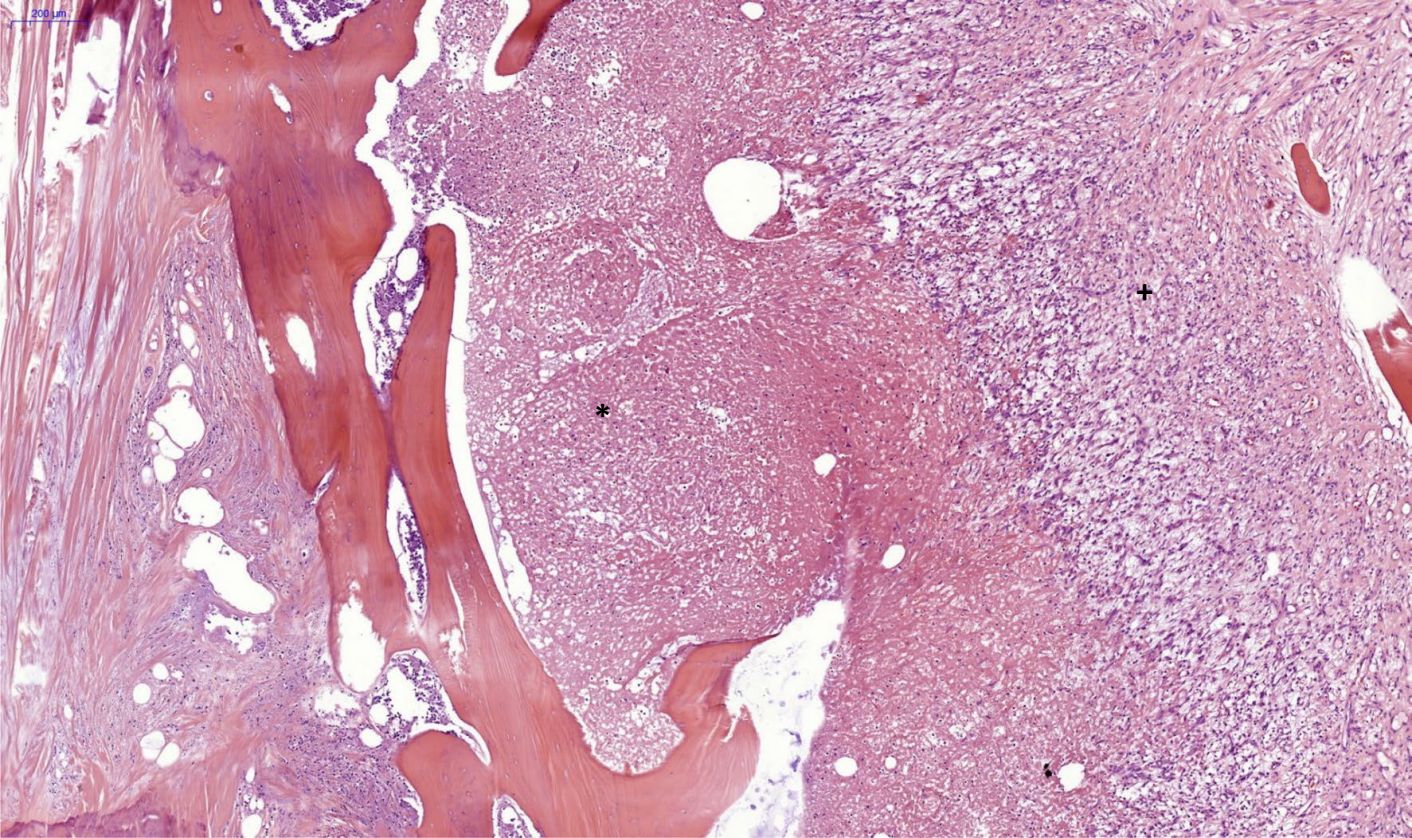
Acute purulent osteomyelitis (*) and transition to a chronic granulating inflammation (+), hematoxylin and eosin stain (H&E), 3× magnification

**Fig. 4:**
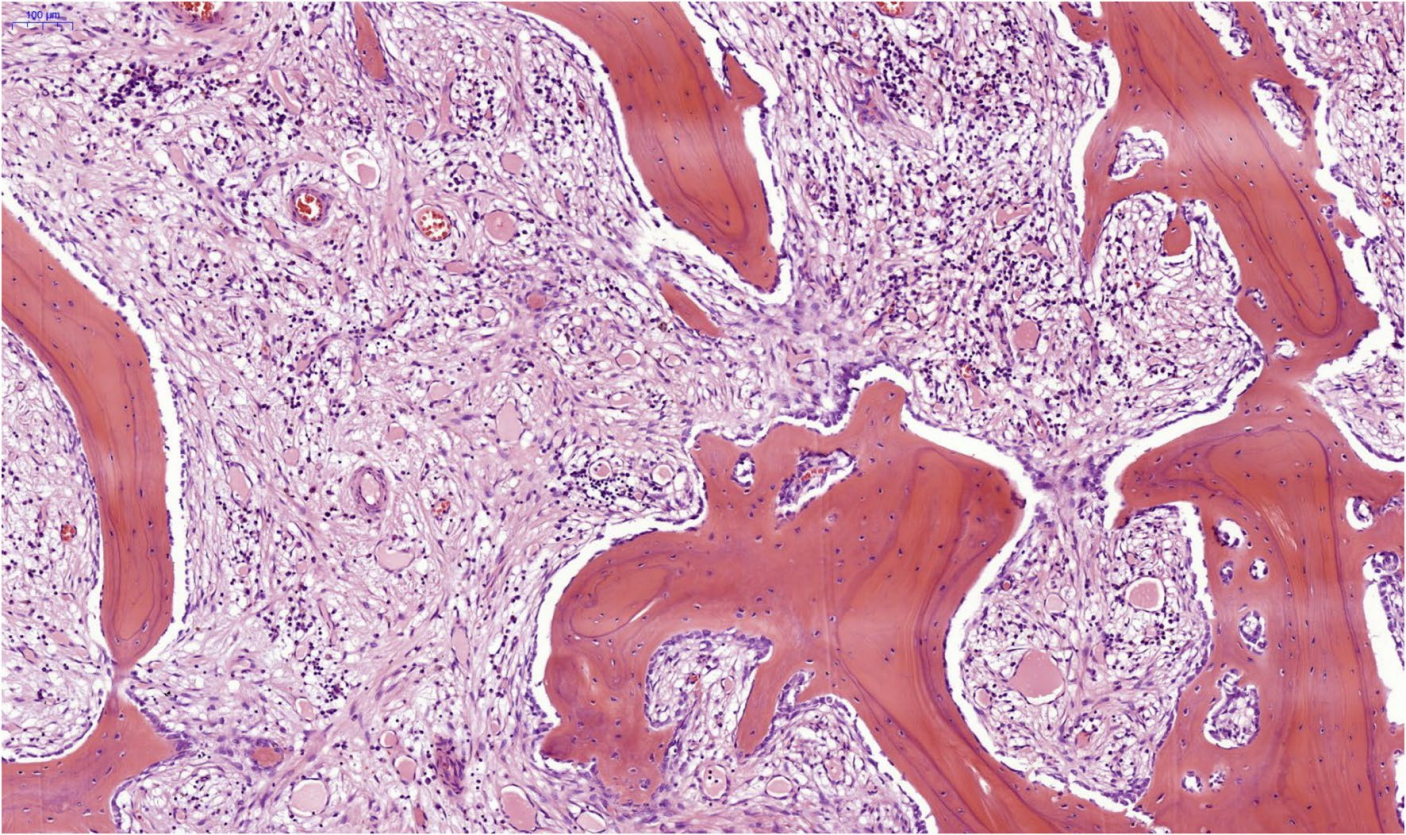
Chronic fibrosing osteomyelitis with new bone formation, H&E, 8×

The median age of the patients in the cohort was 66 (45–81) years and there were 10 females and 37 males. All patients had received a median thoracotomy for open heart surgery. Thirty-seven patients received a coronary bypass, out of which 32 with the left internal mammary artery (LIMA) and one with both mammary arteries. Twenty-seven patients received valve surgery and five received an aortic prosthesis. Forty-seven complete sternal specimens could be histopathologically examined (Figs. 3 and 4).

**Fig. 5:**
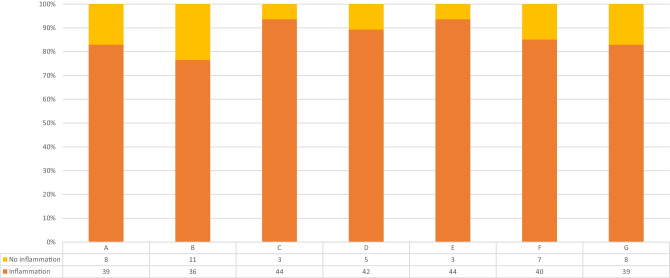
Descriptive results from model 1

Two models were established for the statistical analysis of the histological findings (Table 1). In the first model, the sections were divided according to the presence or absence of bone infection. The results showed the presence of inflammation in 76.6 to 93.6% of the specimens, depending on the section (Fig. 5). Age and sex had no significant impact on the development on bone inflammation, while the body mass index showed a statistical correlation, but only in sections A (*p*=0.004), C (*p*=0.001), and E (*p*=0.002, Student’s *t* test). Smoking, alcohol consumption, diabetes mellitus and insulin intake, myocardial infarction, heart failure, endocarditis, American Society of Anesthesiologist (ASA) classification, coronary bypass surgery, heart valve surgery, and prosthetic aortic replacement proved to have no significant influence on the development of bone inflammation (Fisher’s exact test). Thirty-two patients from the cohort received a coronary bypass using the LIMA. When considering all cases, LIMA harvest had a significant impact only on section A (*p*=0.005, Fisher’s exact test). Furthermore, we compared in the LIMA harvest subcohort the sections from the left side (A, D, F) with the ones from the rights side (B, E, G) and found no significant differences between sections A versus B (*p*=0.25), D and E (*p*=1), and F and G (*p*=0.068, Fischer’s exact test). When comparing the manubrium sections (A and B) with the corpus sterni sections (D, E, F, G) using Fischer’s exact test, we found in this model no significant difference.

**Fig. 6:**
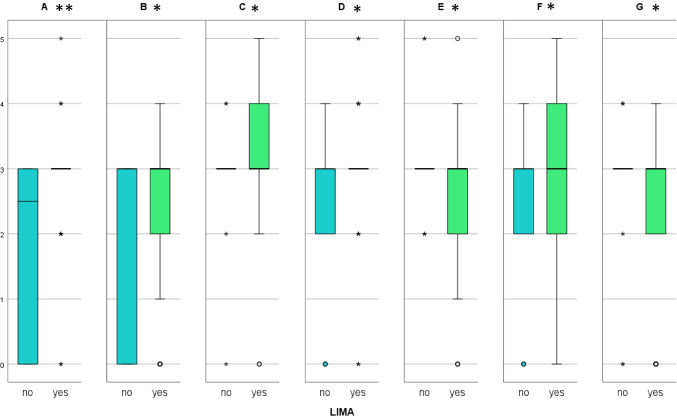
Results from model two, split according to LIMA harvest. *means *p*>0.05, **means *p*<0.05

In the second model, the different levels of bone inflammation were evaluated using a 0- to 5-point score (Table 1). All sections showed a median of 3 points. The cumulative score for the left side was not significantly different than the score of the right side (*p*=0.218, Cohen’s Kappa). When considering only the patients who received a LIMA bypass, there was also no significant difference between the two scores from the left and right side (*p*=0.174, Cohen’s Kappa). While comparing each section with the contralateral one in the LIMA subgroup, section A showed a higher score than section B (*p*=0.027, *χ*^2^), section F higher than section G (*p*=0.015 *χ*^2^), while sections D and E showed no significant difference in scores (*p*=0.14 *χ*^2^) (Fig. 6). Like in the first model, smoking, alcohol consumption, diabetes mellitus and insulin intake, myocardial infarction, heart failure, endocarditis, ASA classification, coronary bypass surgery, heart valve surgery, and prosthetic aortic replacement proved to have generally no significant influence on the bone inflammation score (*χ*^2^). The LIMA harvest had a significant influence on the score only in section A (*p*=0.031, *χ*^2^). When comparing the cranial to the caudal sections in this model, we found significantly less inflammation in section A compared to D (*p*=0.001) and F (*p*=0.006, Cohen’s Kappa). On the right side, there was significantly less inflammation in the sections B compared to E (*p*=0.007 Cohen’s Kappa), while section G (*p*=0.947, Cohen’s Kappa) showed no significant difference.

## Discussion

We hereby present a novel pathological study of sternal osteomyelitis after cardiac surgery. The histological sections showed an overwhelming dissemination of the inflammation across all anatomical parts and across both hemisternums.

DSWI implies the extension of the infection beyond the muscle fascia. These infections usually extend into the anterior mediastinum and involve the soft tissues (skin, subcutaneous tissue, muscle, thymus, pleura, pericardia) and the bone [4]. The development of DSWI may occur due to a combination of factors, which could be classified into bacterial contamination, insufficient blood supply, patient comorbidities, and surgical circumstances [6]. The bone involvement may occur therefore due to the contamination with highly pathogen bacteria like *Staphylococcus aureus*, but also due to less pathogen germs like coagulase-negative staphylococci [3]. While a well-perfused cancellous bone in a healthy individual would resist bacterial contamination, this could not be the case in devascularized bone in a multimorbid patient. Whereas the infection may begin at the level of the soft tissues or within a retrosternal hematoma, the involvement of the defenseless bone will complicate the course of the disease. Not all DSWIs imply a sternal osteomyelitis. If the soft tissue infection is treated promptly and the collections are drained, a secondary closure of the sternum and the soft tissues is possible [7]. On the other hand, once the bone is involved in the infection with transversal fractures and development of sequester, the soft tissue infection will be maintained indefinitely by the SO. In these cases, a radical sternectomy is necessary to treat the infection [5].

Patients with DSWI not only have a heart condition, but mostly have other comorbidities like diabetes mellitus, obesity, or occlusive peripheral arterial disease, which further complicate the DSWI treatment. A swift treatment of the infection is therefore the key to successful treatment. By removing the infected sternal bone en bloc, our team facilitated for the first time the examination of the whole sternum in 47 patients. The results showed that the inflammation is extended to most of the sternal bone, from the manubrium to the xyphoid and from the sternotomy margin to the rib cartilages. The more precise examination using different degrees of bone inflammation, from fibrosis to acute purulent osteomyelitis, showed a ubiquitous distribution of the different types of inflammation, without significant differences between the two hemisternums. When considering the anatomical parts of the sternum, a tendency towards higher degrees of inflammation of corpus sterni was observed, especially on the right side. With the first model this finding could not be confirmed, all anatomical parts being comparably affected by inflammation. This finding has a particular surgical importance. The manubrium stretches laterally towards the first and second ribs and is wider than the caudal sternum. The partial sternectomy does not usually reach the first and second ribs, and according to our current findings it is probable to leave infected bone in place. Even if this lateral bone and the rib joints will not have a purulent infection, it is with high probability infected and should be resected to avoid infection relapse.

The harvest of the LIMA for coronary artery bypass is common and offers improved long-term results [8]. Several anatomic studies have shown different anatomical patterns of the internal mammary artery branches to the sternum and intercostal spaces. The common recommendation is the ligation of the branches close to the main trunk (skeletonization), in order to preserve the collateral circulation [9, 10]. Harvesting the main source of blood supply to the sternum has been shown in different studies to decrease the vascularization, using laser doppler Flowmetry and remission spectrometry [11] or infrared thermography [12]. The skeletonization of the LIMA was proven in a meta-analysis to decrease the incidence of sternal wound infections [13]. Kamiya et al. have shown a drop in retrosternal microcirculation using laser doppler Flowmetry and remission spectrometry, with a significant difference between skeletonized and pedicled LIMA [14], while a similar study proved only the drop in microcirculation with similar results for both pedicled and skeletonized LIMA cases [15]. Using bone scintigraphy, Korbmacher et al. found no perfusion impairment on the LIMA side, although the contralateral side showed an increase in perfusion as a compensatory sign. A similar study in patients with BIMA showed a significant drop in sternal perfusion only in the diabetic subgroup [16], which could be explained by the missing redistribution of circulation in patients with microangiopathy. All this data points to the idea that the development of SO is a multifactorial process, where contamination, blood supply, and comorbidities play a collective role in the pathogenesis. In our study, when considering all examined specimens, LIMA harvest had a significant influence on the level of inflammation on the left side of the manubrium in both models. When analyzing the two hemisternums in the 32 patients with LIMA harvest, the left side of the manubrium showed again significantly more inflammation, but only in the second model. These limited findings could confirm the theory that the sternal devascularization plays a key role in the development of DSWI. On the other hand, the involvement of the right side, where the mammary artery was not harvested, indicates that the pathogenesis of SO involves other factors besides the alteration of blood supply. One theory would be that the devascularized bone is first infected and the infection spreads to the healthy bone over time, facilitated by the presence of foreign bodies (osteosynthesis wires), infected fluid collections, and infected soft tissues.

We further a univariate analysis of the risk factors known to favor the development of surgical site infections and DSWI, like patient-related factors (smoking, alcohol consumption, diabetes mellitus and insulin intake, myocardial infarction, heart failure, endocarditis, ASA classification) or surgery-related factors (coronary bypass surgery, heart valve surgery, and prosthetic aortic replacement) [6], without finding a statistical correlation. As opposed to LIMA harvest, these factors have an overall impact on DSWI and therefore are not expected to have an impact on one specific anatomical region or side of the sternum.

While multiple case reports and case series describe the primary osteomyelitis, which occurs mostly in children and in immunosuppressed patients with common germs like *Staphylococcus aureus* or with atypical germs like Mycobacterium tuberculosis [2, 17–22], the literature is very restrictive in describing the pathology of secondary SO, most studies concentrating on the prevention, epidemiology, microbiology, and treatment of DSWI [6, 23].

The DSWI often extends not only to the sternal bone, but also to the rib cartilages [24], where the infection may persist in the case of an incomplete debridement. After the en bloc resection, we resect the protruding ribs and the sternoclavicular joints on each side. These rib parts could not be systematically examined, which might present a limitation of our study and a direction to further research, in order state the exact extent of the infection beyond the sternal bone.

## Conclusion

Our study proved that the deep sternal wound infection may cause a ubiquitous inflammation of the sternal bone. The decision to preserve or to radically resect the sternum should take into consideration the clinical aspect of the bone and the histopathological findings. If bone preservation is indicated, the patient should be informed about the risk of an acute or chronic infection relapse.

The harvest of the left mammary artery may contribute to the extent and intensity of infection and proves once more that the vascularization plays a key role in the development of DSWI and SO.
